# Long-term survival of monolithic tooth-supported lithium disilicate crowns fabricated using a chairside approach: 15-year results

**DOI:** 10.1007/s00784-023-05023-0

**Published:** 2023-04-21

**Authors:** Angelika Rauch, Lea Lorenz, Sven Reich, Sebastian Hahnel, Anne Schmutzler, Oliver Schierz

**Affiliations:** 1 Department of Dental Prosthetics, University Hospital Regensburg, Franz-Josef-Strauß-Allee 11, 93053 Regensburg, Germany; 2 Department of Prosthodontics and Materials Science, University of Leipzig, Liebigstr. 12, 04103 Leipzig, Germany; 3 Department of Prosthodontics and Biomaterials, Centre of Implantology, Faculty of Medicine, RWTH University Hospital, Pauwellsstr. 30, 52074 Aachen, Germany

**Keywords:** CAD/CAM, Clinical trial, Dental ceramic, Lithium disilicate, Milling, Self-adhesive resin cement, Single crown

## Abstract

**Objectives:**

To investigate the clinical performance of chairside fabricated tooth-supported posterior single crowns from lithium disilicate ceramic.

**Materials and methods:**

Thirty-four crowns (IPS e.max CAD, Ivoclar Vivadent, Schaan, Liechtenstein) were inserted between 2006 and 2007 and again evaluated after 15 years. Survival and success rates were calculated according to Kaplan–Meier, and the quality of the crowns was evaluated by using modified United States Public Health (USPHS) criteria.

**Results:**

Twenty-two crowns were available for recall; six patients were defined as dropouts. The mean observation period was 15.2 years (± 0.2). Six failures occurred (1 technical/5 biological) resulting in a survival rate of 80.1%. The success rate was 64.2%. The roughness of the crowns increased (*p* = 0.021) and the majority of adhesive gaps were discolored (*p* = 0.001) in comparison to baseline. The color, tooth, and crown integrity remained stable over the follow-up period (*p* ≥ 0.317).

**Conclusion:**

The fabrication of tooth-supported lithium disilicate crowns using a chairside approach yielded acceptable long-term survival and success rates. Due to discoloration, the long-term use of dual-cure self-adhesive resin cements might result in unpleasing esthetic results.

**Clinical relevance:**

The performance of posterior lithium disilicate single crowns revealed excellent to good clinical quality and an acceptable number of events after 15 years of clinical service.

## Introduction

Lithium-based glass ceramics have gained in reputation over recent years and are highly favored by dentists for their application as single crowns or fixed partial dentures (FPD) [1, 2]. Due to their popularity, manifold products related to lithium-(X)-silicate ceramics are available that can be classified either as lithium disilicate, lithium silicate, lithium (di) silicate, or lithium aluminosilicate disilicate. These materials differ in their crystalline and glass phase fractions and their mechanical properties. The first machinable lithium-based glass ceramic for milling (CAD/CAM), a lithium disilicate ceramic, was developed by Ivoclar Vivadent (Schaan, Liechtenstein). After conventional crystallization, it consists of 63 vol% Li_2_Si_2_O_5_, 7 vol% Li_3_PO_4_, 1 vol% cristobalite, and 30 vol% residual glass. A pressable lithium-based glass ceramic developed by the same company is also available that contains lower fractions of Li_2_Si_2_O_5_ and cristobalite, yet 1 vol% Li_2_SiO_3_ fractions and higher fractions of residual glass [3]. Both materials can be used to fabricate partial restorations, single crowns, or 3-unit FDPs, although their indication range is limited to FDPs that extend up to the second premolar as the terminal abutment. The machinable lithium-based glass ceramic is utilized in digital workflows and even chairside approaches. The benefits of optical impression and CAD/CAM workflow in contrast to approaches with conventional impression taking and the lost-wax technique are controversial. Preferences are likely to depend on the practitioner’s individual focus regarding, e.g., the duration of the impression taking, laboratory working time, or digital affinity [4, 5]. Nonetheless, no significant differences in terms of crown fit have been observed [6].

Another relevant parameter for decision-making in restorative dentistry is the clinical performance of the restorative materials. For pressable lithium disilicate ceramics, few long-term results are available. Edelhoff et al. examined single-tooth restorations in patients with moderate to severe tooth wear. After a mean observation period of 8 years, all of the inserted restorations were still in situ [7]. Garling et al. examined 3-unit FDPs with the majority of FDPs replacing molars. After 15 years of clinical service, they observed a survival rate of 48.6% and a success rate of 30.9% [8]. In these studies, all restorations had been processed using a conventional workflow. Regarding machinable lithium disilicate ceramics, data from our patient cohort have already been published addressing an observational period of 10 years for milled lithium disilicate single crowns [9–11]. Aziz et al. investigated metal-ceramic crowns and milled monolithic lithium disilicate crowns after 6 years of clinical service and observed that the survival and success rates were higher for the monolithic restorations [12]. To the authors’ knowledge, no other long-term results for monolithic lithium disilicate restorations are available [13].

The aim of this investigation was to add information on the clinical performance of monolithic lithium disilicate ceramic single crowns from the aforementioned cohort that were fabricated in a chairside approach, after 15 years of clinical service.

## Material and methods

Thirty-four patients received a crown fabricated from lithium disilicate ceramic (IPS e.max CAD LT, Ivoclar Vivadent) in a chairside approach between 2006 and 2007. Seven patients received two crowns; however, just one crown per patient was randomly selected for statistical evaluation (*n* = 34; premolar/molar 8/26; 17 teeth endodontically treated). The exclusion criteria were xerostomia, temporomandibular disorders, or pregnancy. The inclusion criteria were abutment tooth vital or with successful endodontic treatment, existing adjacent teeth, and pocket depths ≤ 3.5 mm.

The requirements of the Helsinki Declaration were followed and the patients gave their signed informed consent. The study was approved by the local ethical committee (no. 103–2006).

All crowns were fabricated by experienced and calibrated dentists; 20 patients were treated in a university setting and 14 patients in a private dental practice. For chairside fabrication of the crowns, an infra-red camera (Cerec 3, Software version 2.9, Sirona, Bensheim, Germany) and a milling unit (Cerec 3 milling unit, Sirona) were used. Try-ins of the crowns were carried out with the material in its lithium metasilicate state (blue), and corrections were made if necessary. The restorations were stained and glazed (IPS e.max CAD Crystall./Glaze Paste, Ivoclar Vivadent) and crystallized (Programat CS, Ivoclar Vivadent). For self-adhesive cementation, the teeth were cleaned with pumice and hand instruments. The intaglio surface of the crowns was etched with hydrofluoric acid (IPS Ceramic Etching Gel, Ivoclar Vivadent) for 20 s and a silane coupling agent was applied for 60 s (Monobond S, Ivoclar Vivadent). The restorations were inserted with a dual-cure self-adhesive resin cement (Multilink Sprint, Ivoclar Vivadent).

The crowns were examined at baseline, after 6 months, 1 to 6 years, 10 years, and 15 years. The primary outcome variables were survival, i.e., the crown was still in situ, and success, which also considered complications. Technical complications included the occurrence of crown fractures, loss of retention, or chipping of the ceramic. Biological complications included carious lesions below the crown margin, abutment fractures, or endodontic interventions. The clinical quality of the single crowns was assessed by using the modified US Public Health Service (USPHS) criteria [11]. The recall was conducted by two independent dentists who were not involved in the initial treatment procedures. In case of dissent, consent was achieved by discussion.

Statistical analyses including Kaplan–Meier analysis, log-rank tests, and Wilcoxon signed-rank tests were conducted (IBM SPSS Statistics 28, IBM, Armonk, NY, USA). The level of significance was set to *p* < 0.05.

## Results

After a mean observational time of 15.2 years (± 0.2 years), 22 patients (58.9 years, 72% female) were examined. Six patients were defined as dropouts since three patients were deceased and three patients could not be contacted by mail or telephone. Six crowns with their respective teeth had also already been removed.

### Clinical quality (modified USPHS criteria)

**Fig. 1:**
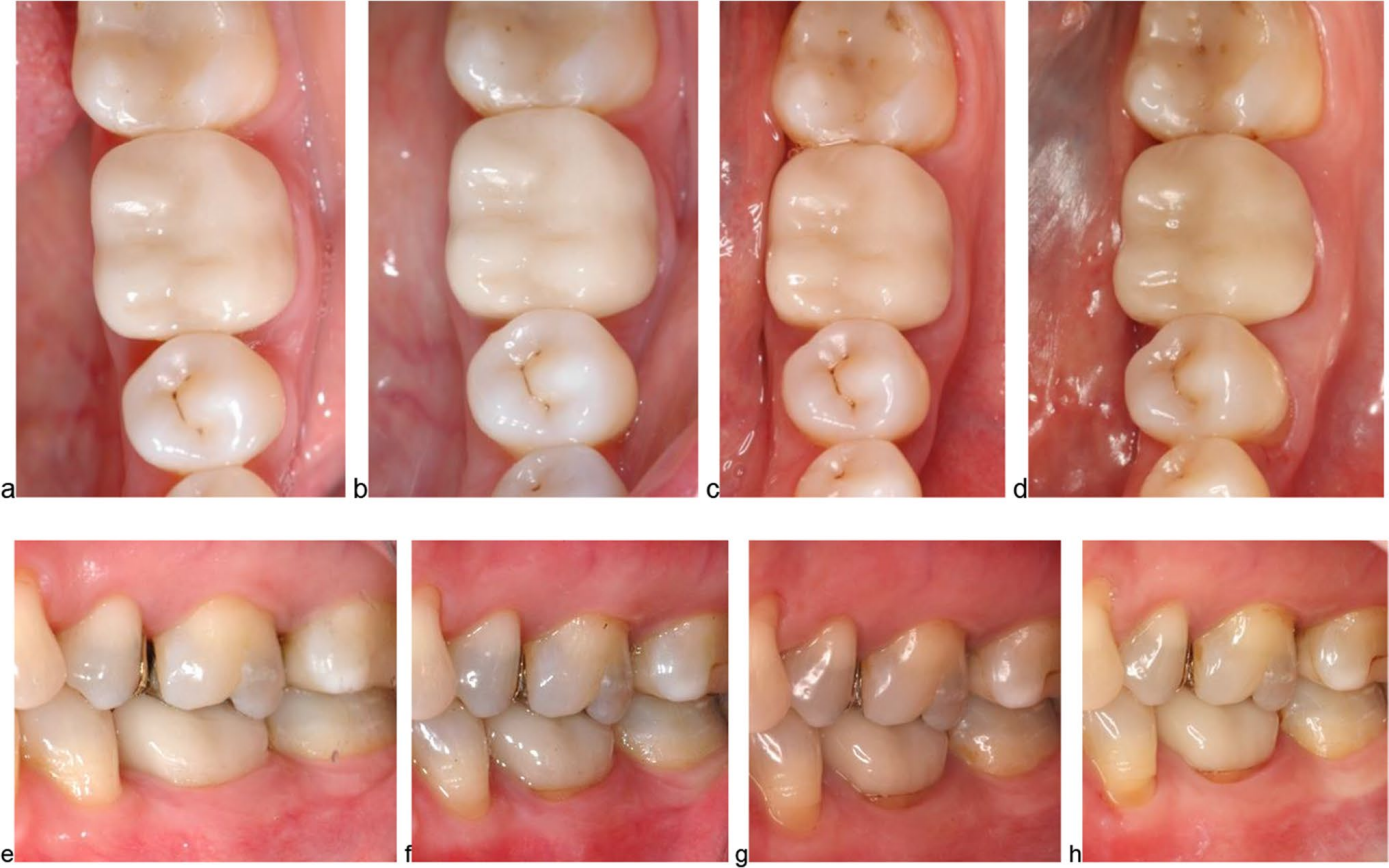
Clinical pictures of a lithium disilicate study crown (FDI 36) at baseline (**a**, **e**), at 5-year recall (**b**, **f**), at 10-year recall (**c**, **g**), and at 15-year recall (**d**, **h**); occlusal view (**a**–**d**), vestibular view (**e**–**h**)

**Fig. 2:**
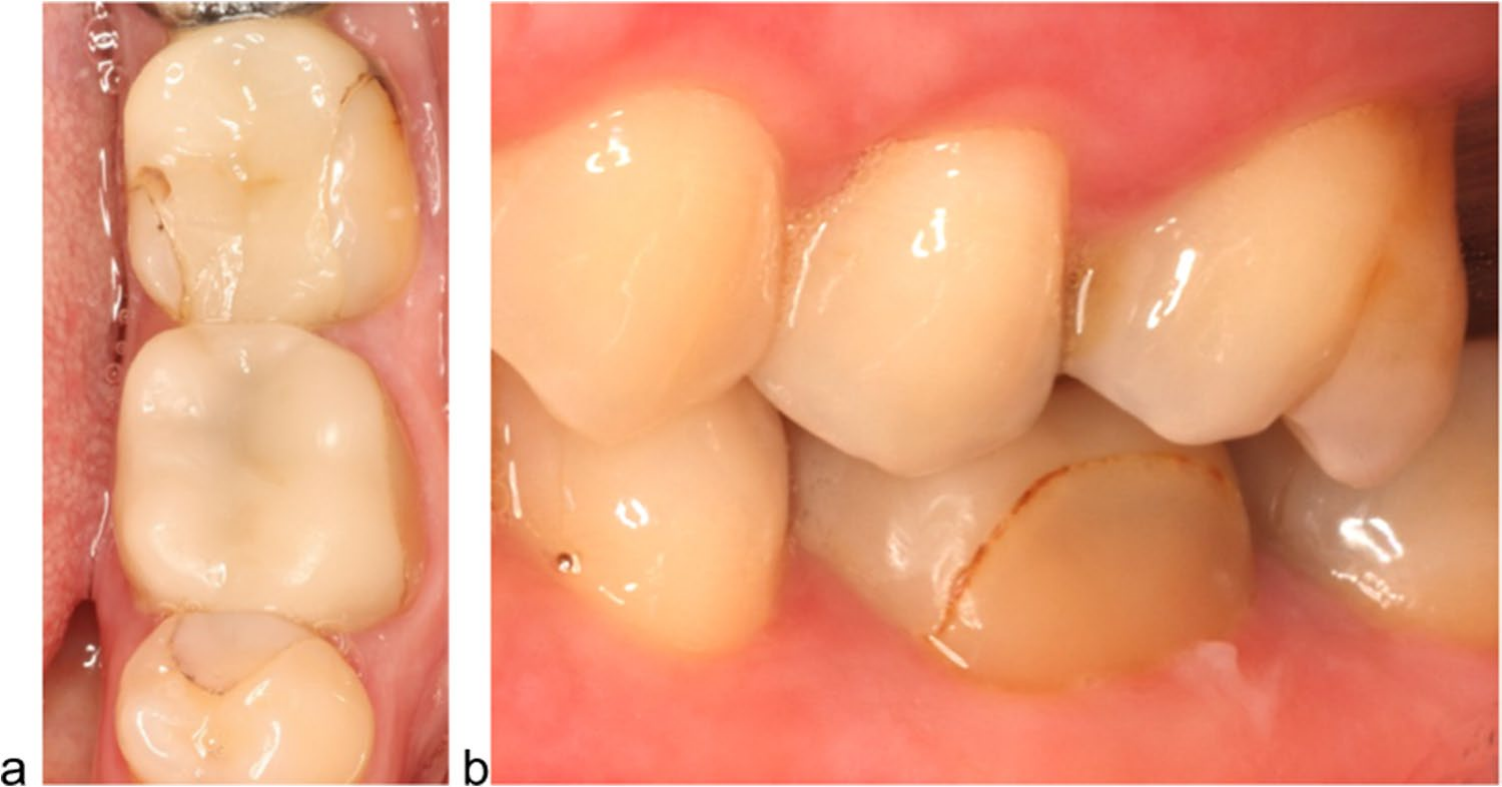
At 15-year recall, the vestibular part of a crown presented a direct resin-based composite restoration (**a** occlusal view; **b** vestibular view). A fracture of the crown occurred after an accident had happened at 11.1 years of clinical service

After 15 years, the quality of the surface changed significantly from A1 scorings to A2 scorings (Wilcoxon signed-rank test: *p* = 0.021). The A2 scoring for surface characterizes a rough surface that can be polished. The color of the crowns was evaluated as consistent comparing the two timepoints (Wilcoxon signed-rank test: *p* = 0.480, Fig. 1a–d). For the adhesive gap, a lower percentage of crowns was rated with an A1 and more crowns were scored with A2 or B; a scoring of B refers to a discolored gap that cannot be polished (Wilcoxon signed-rank test: *p* = 0.001, Fig. 1e–h). The integrity of the abutment tooth changed slightly for one abutment tooth from A1 to B, since a wedge-shaped lesion was observed after 15 years. After 11.1 years, one patient had an accident that caused a fracture of the vestibular part of the crown. The restoration was repaired with a resin-based composite in a direct approach, yet the original shape of the crown could not be preserved (Fig. 2). This event was counted as a technical complication and resulted in a C scoring for crown integrity. Neither the scoring for the integrity of the abutment tooth nor for the crown significantly changed over the observational period (Wilcoxon signed-rank test: *p* = 0.317). One abutment tooth received endodontic treatment after 1.1 years and was asymptomatic afterwards. This event was counted as a biological complication and led to a D rating (Wilcoxon signed-rank test: *p* = 0.546). None of the patients had complaints regarding the crown, which was a significant improvement in comparison to baseline (Wilcoxon signed-rank test: *p* = 0.014). Three patients reported that they had occasionally observed food residues in connection with the crown, which resulted in B scorings for compliance (Wilcoxon signed-rank test: *p* = 0.157). Detailed information is displayed in Table 1.

**Table 1:**
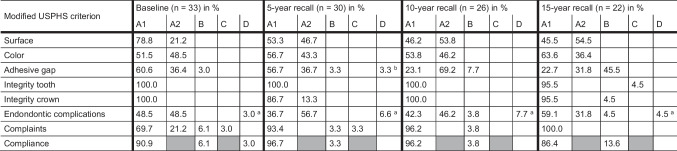
Qualitative assessment of the available crowns according to the modified USPHS criteria (A1 = excellent, A2 = good, B = sufficient, C = insufficient, D = poor)

^a^Endodontic complications: *A1* vital tooth, *A2* successful endodontic treatment before crown restoration, *D* sensitivity change

^b^*Delta* rating: carious lesion below the crown margin

### Biological events

During the 15-year follow-up period, eight biological events were observed. At baseline, one tooth did not react to the sensitivity test. The patient had no complaints. A dental x-ray after 10 years revealed no apical radiolucency, and after 15 years, the reaction to sensitivity testing was slightly positive. This observation was defined as a biological complication at baseline, but was rated with A at the 15-year recall. Another abutment tooth received endodontic treatment after 1.1 years (D rating). The study crown could be removed without damage and successful root canal treatment was performed. Subsequently, the crown was cemented again. For one study crown, a carious lesion below the crown margin had to be removed after 4 years. A resin composite filling was applied. After 6 years, two severe biological complications occurred as one abutment tooth had to be extracted due to an apical infection. Another abutment tooth featured a fracture caused by a carious lesion and had to be supplied with a new crown. At the 7-year follow-up, a longitudinal fracture of the mesial root of a lower molar was detected. The abutment tooth had to be extracted. One patient had already presented various carious lesions near the crown margin during the 2–5-year follow-up period; all lesions were treated with direct fillings. This event was counted as a complication after 24 months. After 10 years, another carious lesion occurred and the study crown had to be replaced, which was counted as a failure but not again as a complication. After 12.7 years, another crown had to be replaced due to a carious lesion affecting the crown margin.

### Technical events

During the 15-year follow-up period, three technical events occurred. One molar crown lost retention after 2 years. As no carious lesion was detected, the restoration was etched with hydrofluoric acid, silanized, and cemented with a self-adhesive resin cement (RelyX Unicem, 3 M, Seefeld, Germany). This event was counted as a complication. After 2.8 years, a fracture of another study crown occurred, which was regarded as a failure. One patient had an accident after 11.1 years and a fracture of the vestibular part of the crown occurred, which was registered as a complication. In emergency dental service, the surface was replaced with a direct filling (Futurabond DC, VOCO, Cuxhaven, Germany; Gradia Direct LoFlo, GC, Luzern, Switzerland; Fig. 2).

### Kaplan–Meier analysis

**Fig. 3:**
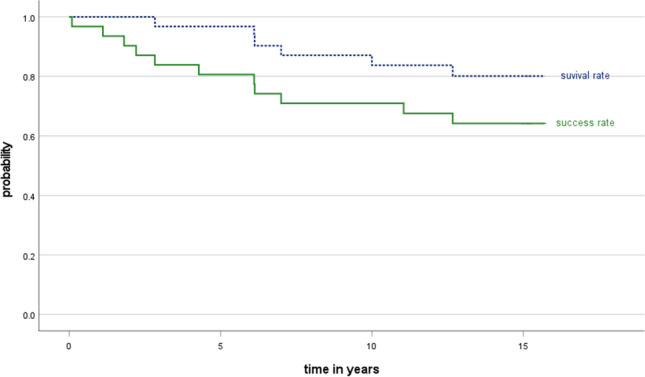
Kaplan–Meier analysis of the survival and success of the monolithic lithium disilicate crowns after 15 years of observation (*n* = 22)

The survival rate after 15 years was 80.1% [95% CI 56.8; 94.4]. Success, which includes both failures and complications, was calculated as 64.2% [95% CI 47.1; 81.3] including all biological and technical events (Fig. 3). No significant differences could be identified when the character of the abutment tooth (premolar/molar; log-rank *p* = 0.120), endodontic treatment of abutment teeth (log-rank *p* = 0.699), or the setting of the dental treatment (university/private dental practice; log-rank *p* = 0.233) were taken into account (Table 2).

**Table 2:** Overview over failures (F) and complications (C); success rates after 15 years of clinical service; premolar (PM), molar (M)

Event	Number of events	Event	Abutment tooth	Abutment tooth sensitivity at time of insertion		Setting	
				Positive sensitivity (*n* = 17)	Succesfully endodontically treated (*n* = 17)	University (*n* = 20)	Private practice (*n* = 14)
Crown fracture	1	F	M	1	0	0	1
Apical infection	1	F	M	0	1	1	0
Abutment fracture	1	F	M	0	1	1	0
Root fracture	1	F	M	0	1	1	0
Carious lesion near crown margin	2	F	PM + M	0	2	2	0
Carious lesion near crown margin	1	C	M	1	0	1	0
Change of sensitivity	2	C	M	2	0	1	1
Loss of retention	1	C	M	2	0	1	0
Chipping	1	C	M	1	0	0	1
Total	11		1 PM; 10 M	7	5	8	3
Success rate after 15 years	64.2%		85.7%, 56.5%	68.8%	59.3%	55.0%	76.9%
Log-rank test			*p* = 0.120	*p* = 0.699		*p* = 0.233	

## Discussion

After 15 years of clinical service, six single crowns had been removed and six patients were not available for recall. The survival rate was 80.1% and the success rate was 64.2%. No significant differences were observed whether the abutment tooth was a premolar or molar, if endodontically treated teeth, or not in terms of the dental treatment setting. In comparison to the baseline examination, a significantly higher proportion of crowns featured rough surfaces or discolorations of the adhesive gap.

The survival and success rates of the crowns have decreased since the 10-year recall (83.5% and 71%). This was especially due to the occurrence of carious lesions of the abutment teeth. For single crowns, an influence of the restorative material on the occurrence of events has been heterogeneously described in the literature [12, 14]. However, carious lesions are frequently observed even for abutment tooth supplied with metal restorations. Nonetheless, the survival and success rates of the crowns observed in the current study are notably higher than those reported by Garling et al. who observed 3-unit FDPs fabricated from a pressable lithium disilicate ceramic with values of 48.6% or 30.9% [8]. A recent systematic review highlighted that monolithic tooth-supported FDPs fabricated from ceramic might be more affected by failures or complications than single crowns [13]. This conclusion is not surprising since the probability for the weakest link is higher in multi-unit than in single-unit FDPs. Moreover, Garling et al. extended the indication range for multi-unit FDPs fabricated from lithium disilicate ceramics to the molar region, which might also explain the high number of events.

The changes to the surface of the crowns with respect to roughness can be attributed to wear, which is a well-known phenomenon in restorations fabricated from lithium disilicate ceramics. However, the surfaces of all crowns were rated either as excellent (A1) or polishable (A2) even after a period of 15 years, which refers to a clinically acceptable quality. These results correspond to in vitro investigations that observed a roughness of lithium disilicate specimens of approximately 1.6–1.9 µm after pin-on-bloc testing (Ra), which can be polished by using multi-step approaches [15, 16]. Moreover, no relevant surface volume loss was obvious in the present clinical trial since the integrity of the crown was still given. This observation is similar to in vitro investigations that observed wear depths of 130 µm, which cannot be detected by the eye [15].

Relevant changes were observed in the evaluation of the adhesive gap, since 78% of the gaps showed a discoloration that could be polished. Some adhesive gaps were also classified as “not possible to polish.” Even though the application of dual-cure self-adhesive resin cements is recommended in areas with limited or no access for the polymerization light, these materials develop discolorations over time [17], since they contain camphorquinone, benzoyl peroxide, and tertiary amines. However, a recent in vitro investigation reported that discoloration may also occur when amine-free products are applied [18]. Additionally, in the clinical setting, extrinsic factors like beverages, food, or smoking can also have an important impact on discoloration. As an alternative, conventional cements could be applied with IPS e.max restorations when the material thickness is equal to or greater than 1.5 mm. According to a systematic review by Maroulakos et al., comparable survival and success rates independent of the cementation method can be expected [19].

The limitations of this study include the lack of a comparison group and the small number of patients that had been recruited. Moreover, CAD/CAM systems have been continuously improved over the last decade; thus, the improved accuracy and performance of the optical scanners, software, milling machines, and furnaces might produce even better clinical results than observed in the current study. Nonetheless, the strengths of this investigation include its prospective character, the long-term observational period, the evaluation of the restorations by independent dentists, and the study design that included both a university as well as a private practice setting.

## Conclusion

Within the limitation of this prospective trial, tooth-supported single crowns fabricated from lithium disilicate ceramic featured an acceptable performance after 15 years of clinical service. The color, tooth, and crown integrity yielded stable results; yet the long-term use of dual-cure self-adhesive resin cements may result in unpleasing esthetic results. The chairside approach for the fabrication of lithium disilicate ceramic single crowns in the posterior area can be recommended.

## Data Availability

Data available on request from the authors.
